# PlasmoDocking: A User‐Friendly Open‐Source Web Tool for Virtual Screening Targeting *Plasmodium falciparum* Enzymes

**DOI:** 10.1002/jcc.70225

**Published:** 2025-09-04

**Authors:** Fernando Loza Guariero, Eduardo Pantoja de Macedo, Elise Bittencourt de Laia, Joseph Albert Medeiros Evaristo, Geisa Paulino Caprini Evaristo, Fernando Berton Zanchi

**Affiliations:** ^1^ Laboratório de Bioinformática e Química Medicinal, Fundação Oswaldo Cruz Rondônia (LABIOQUIM‐Fiocruz‐RO) Porto Velho Rondônia Brazil; ^2^ Departamento de Química Universidade Federal de Rondônia (UNIR) Porto Velho Rondônia Brazil; ^3^ Departamento de Ciência da Computação Universidade Federal de Rondônia (UNIR) Porto Velho Rondônia Brazil; ^4^ Centro de Estudos de Biomoléculas Aplicadas à Saúde, Fundação Oswaldo Cruz Rondônia (CEBio‐Fiocruz‐RO) Porto Velho Rondônia Brazil; ^5^ Programa de Pós‐Graduação em Biologia Experimental Universidade Federal de Rondônia (UNIR) Rondônia Brazil

**Keywords:** AutodockGPU, docking, multi‐target, *Plasmodium falciparum*, web‐based tool

## Abstract

Virtual screening through molecular docking represents a fundamental computational methodology extensively employed in the identification of therapeutic compounds for malaria and other parasitic diseases. Although numerous software platforms are available, including AutodockGPU, the command‐line interface requirements present significant barriers to non‐specialized users, while multi‐target screening protocols introduce additional complexity in receptor preparation procedures. To address these limitations, we developed Plasmodocking, a comprehensive web‐based platform designed to automate molecular docking simulations against predefined *Plasmodium falciparum* targets (https://plasmodocking‐unir.ecotechamazonia.com.br/). The platform enables users to submit up to 10 molecular structures (.sdf format) for automated AutodockGPU screening against 38 pre‐configured parasite targets, facilitating systematic comparison of binding energies with co‐crystallized ligands. Developed using Python and Next.js, Plasmodocking accelerates malaria drug discovery by enabling simultaneous multi‐target docking simulations within a single experimental framework. The open‐source codebase is available at: https://github.com/LABIOQUIM/PlasmoDocking‐Client.

## Graphical abstract

We present Plasmodocking, a web platform that automates the molecular docking process using Autodock GPU for multi‐targeting Virtual Screening (*N* × *N*). Simply provide up to 10 candidate inhibitor molecules in .sdf format, and the software will execute docking against 38 prepared and validated target enzymes from *Plasmodium falciparum*. At the end, you can view a dashboard with individualized comparisons for each enzyme.

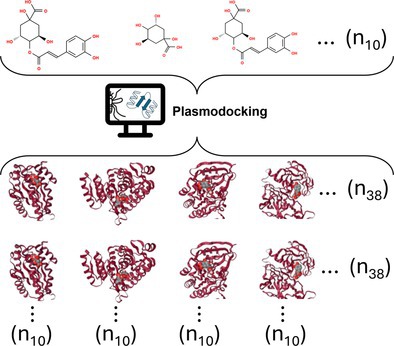

## 1 Introduction

The development of new drugs has increasingly focused on targeting enzymes that are vital to the survival and infectivity of pathogens [1, 2]. This approach, known as rational drug design, aims to identify specific inhibitors (molecules) that can block essential biochemical pathways within the pathogen [3, 4]. By understanding the structure and function of these target enzymes, it may be possible to design or to find in the databases molecules that bind precisely to the active site, thereby inhibiting their activity and disrupting the pathogen's life cycle. This method not only enhances the specificity and efficacy of potential drugs but also reduces the likelihood of off‐target effects and adverse reactions.

As biological information and the number of molecules in active chemical databases have been doubling every year, in silico bioinformatics approaches have become essential. These methods provide a high‐throughput, cost‐effective solution for mining bioactive molecules from these growing databases [5, 6].

Among these techniques, Virtual Screening (VS) by molecular docking is one of the most widely recognized [7]. It involves searching for multiple bioactive molecules using high‐performance computers and software that simulates interactions between molecular targets and small molecules, which can potentially become drug candidates. The study of molecular interactions through molecular docking is crucial in the search for small molecules, using data based on the structures of ligands and receptors.

This approach aids in the design of new drugs by analyzing the interaction bonds and the stability of the complexes formed between enzyme targets and ligands [8, 9, 10]. Currently, there are numerous protein‐ligand molecular docking tools available, such as Autodock4 [11], Autodock Vina [12, 13], Glide [14], MOE [15], and Dockthor [16]. Most of these tools rely on CPU (Central Processing Unit) processing. However, with the advent of parallel processing on GPU (Graphics Processing Units), new docking software like AutodockGPU [17] has emerged to accelerate this process. AutodockGPU is essentially a GPU‐optimized version of Autodock4.

Despite these advancements, users often face challenges when conducting experiments with multiple ligands or targets using AutodockGPU, as it still operates via command line. Additionally, the preparation and standardization of targets, the cost of high‐performance computers, and the steep learning curve for interpreting results add to the complexity of this approach.

To address these challenges, we have developed a new web tool that automates VS processes using AutodockGPU for all 38 *P. falcipa*rum target enzymes available in the Protein Data Bank—PDB [18, 19]. This tool, which we have named Plasmodocking, simplifies the process, making it more accessible and efficient for researchers.

## 2 Computational Methods

The implementation follows the classical web application model, where a client terminal consumes HTML content (front‐end) provided by a server over the internet (back‐end). The application was named Plasmodocking and uses AutodockGPU, Python 3.5, Anaconda, PostgreSQL, Celery with RabbitMQ as a backend. All running on a Linux architecture. The front‐end is built using Django, Next.js, CSS, and HTML5. The application is accessible at https://plasmodocking‐unir.ecotechamazonia.com.br, and users are required to log in to access the website. Each run in Plasmodocking supports up to 10 ligands, and users can disconnect from the server and reconnect later to check the results.

### 2.1 Redocking and Target Preparation

In order to validate the virtual screening experiments, redocking was carried out using the MGLTools graphical interface to prepare the receptors, the ligands, and the AutodockGPU in traditional mode, command line. The grid box centers were set thereabout the original ligands/inhibitors present in each structure, and their size was adjusted individually to better fit the surrounding structures. Some adjustments were made individually to each grid to ensure that the redocking protocol achieves the lowest possible RMSD. Subsequently, the ligands were removed from the structures and then subjected to a protocol using the previously defined individual grid applied in the Lamarckian genetic algorithm in semi‐flexible default settings, configured with 50 independent runs per ligand, and all other settings were kept at default [11]. Initially, 205 pre‐selected proteins of *Plasmodium falciparum* strain 3D7, without any mutations, were listed. After exclusion of repeats and filtering for lower resolution, 38 different proteins were standardized and incorporated into Plasmodocking. The best redocking parameters for each protein are detailed in Table 1.

**TABLE 1:** Redocking parameters of the 38 crystallized ligands on their respective target molecules.

*N*	PDB ID	Cryst. Ligand	Grid center [Å] (size) [Å]			RMSD [Å]	ΔG (kcal/mol)
			*X*	*Y*	*Z*		
01	1CJB	POP	59.285 (26)	33.462 (33)	75.224 (49)	1.09	−4.89
02	1G1G	MTI	8.147 (30)	5.374 (31)	91.635 (32)	0.52	−8.57
03	1LF2	R37	31.829 (42)	33.951 (46)	14.300 (39)	0.98	−9.75
04	1LYX	PGA	20.766 (25)	1.407 (29)	7.395 (30)	2.34	−2.40
05	1P9B	IMO	30.987 (35)	68.449 (37)	24.892 (35)	1.95	−8.48
06	1Q4J	GTX	9.867 (32)	0.219 (38)	20.350 (34)	1.35	−5.97
07	1T26	GBD	25.339 (31)	19.289 (31)	5.559 (35)	0.03	−4.64
08	1VYQ	DUX	38.113 (42)	10.902 (31)	−9.252 (34)	3.04	−8.62
09	2I7C	AAT	16.742 (47)	115.486 (30)	27.654 (33)	0.43	−15.11
10	2WWF	TMP	8.321 (31)	12.705 (25)	5.742 (30)	0.56	−7.55
11	3BWK	C1P	−8.868 (45)	24.651 (44)	9.993 (36)	1.97	−9.38
12	3PR3	F6P	14.451 (30)	12.159 (32)	13.696 (30)	1.18	−4.99
13	3QGT	CP6	28.409 (31)	5.895 (31)	58.672 (35)	0.04	−8.53
14	3QS1	006	27.528 (47)	9.829 (31)	4.92 (37)	0.85	−11.15
15	3QVI	K95	14.777 (40)	16.814 (35)	3.820 (32)	2.24	−5.15
16	3SL1	FB6	38.572 (35)	16.931 (31)	24.030 (30)	1.36	−4.79
17	3UJ8	SFG	24.721 (53)	18.064 (41)	17.545 (39)	0.15	−9.82
18	3VI2	HMZ	20.517 (32)	33.990 (34)	13.690 (30)	2.87	−4.51
19	4J56	FAD	−31.193 (70)	108.475 (38)	197.785 (34)	0.79	−13.67
20	4J75	TYM	21.780 (31)	12.940 (49)	−0.060 (30)	0.45	−13.07
21	4JFA	TRP	15.178 (27)	16.624 (28)	21.443 (30)	0.58	−7.25
22	4PG3	KRS	−46.774 (34)	35.197 (32)	−9.363 (31)	0.54	−9.19
23	4TR9	38D	−8.496 (35)	14.834 (34)	26.615 (44)	2.03	−5.59
24	4ZCS	CDC	12.825 (35)	39.857 (40)	72.148 (53)	1.09	−9.68
25	5BOO	ORO	−16.482 (52)	−7.432 (50)	−5.258 (38)	0.35	−8.72
26	5BOO^a^	D65	−28.385 (45)	−6.630 (45)	−13.310 (45)	0.85	−9.54
27	5JAZ	LC5	1.088 (33)	13.459 (38)	18.841 (36)	0.14	−9.60
28	6FBA	D48	64.164 (35)	57.897 (35)	−124.554 (30)	3.15	−6.13
29	6JW9	E64	−10.737 (38)	14.556 (37)	−39.487 (39)	2.53	−4.88
30	6R8G	JUT	−1.185 (27)	55.352 (33)	−24.911 (40)	4.47	−6.55
31	7DIA	YMZ	−53.571 (39)	−1.080 (37)	2.016 (35)	0.02	−5.88
32	7DPI	B79	−7.828 (44)	6.204 (37)	−26.576 (47)	0.86	−9.66
33	7MXY	HV6	152.087 (41)	171.866 (30)	141.039 (30)	1.01	−7.01
34	7QB7	9X2	44.775 (32)	4.933 (35)	131.539 (37)	0.80	−13.11
35	7ROR	69X	9.915 (35)	3.063 (31)	54.756 (50)	0.71	−10.53
36	7TBC	I01	−44.410 (36)	32.366 (30)	7.350 (33)	0.54	−12.64
37	7ZGS	PRA	23.285 (30)	−0.536 (40)	5.861 (30)	3.71	−5.04
38	8EWZ	X01	14.905 (29)	118.716 (35)	15.157 (49)	1.02	−7.19

*Note:* The ligand codes for each PDB record are reported. Grid coordinate centers represent the geometric center and size of the grid. The RMSD and ΔG values correspond to the best position and the best binding free energy obtained for the redocking process of ligands with Plasmodocking.

^a^ Redocking in an allosteric site.

### 2.2 Plasmodocking Interface and Use Case

**FIGURE 1:**
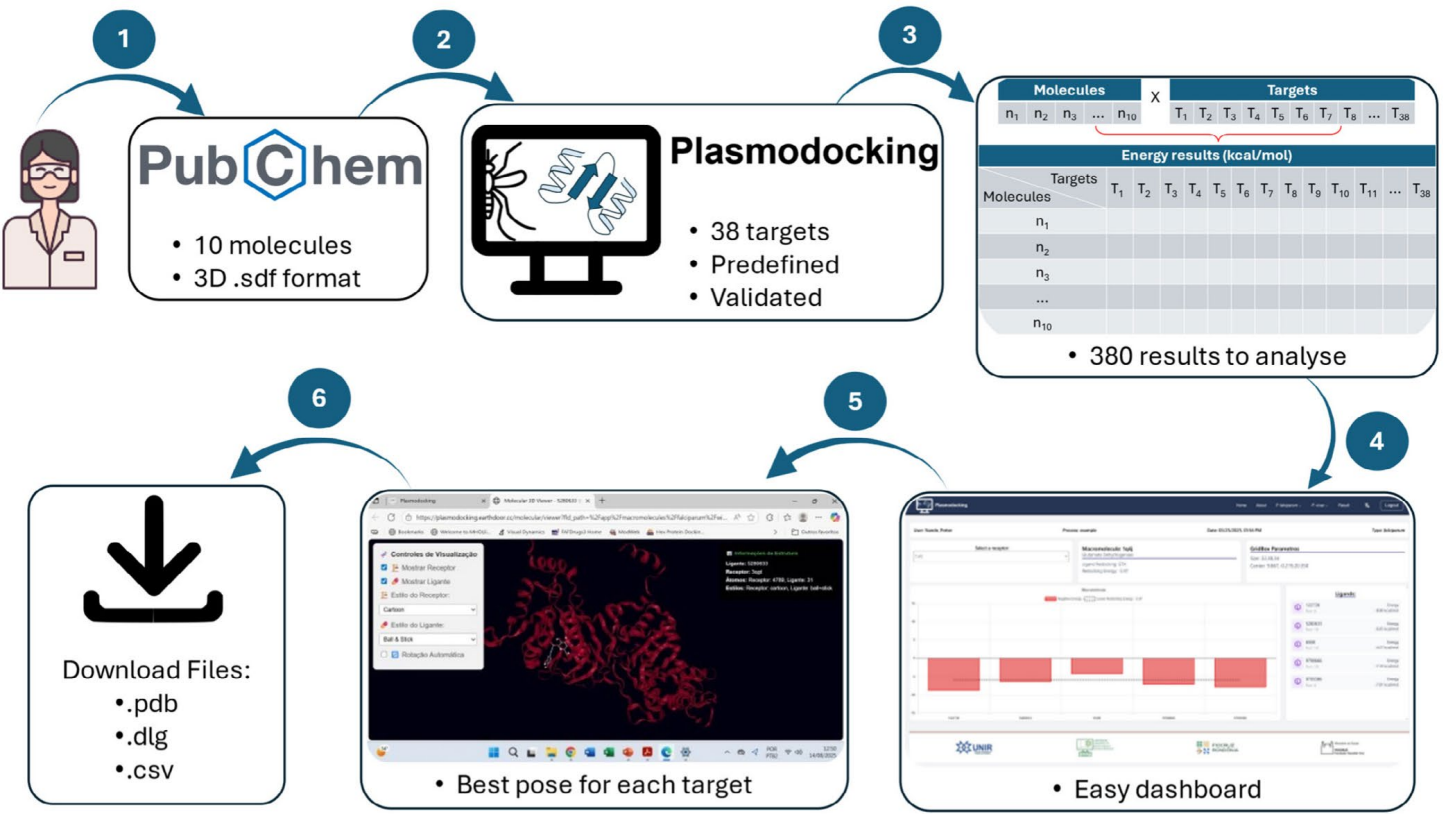
Plasmodocking pipeline. (1) The user selects up to 10 molecules from PubChem (or another database) in 3D .sdf format. (2) The user submits this .sdf file to Plasmodocking. (3) The software will prepare the ligands and perform docking against 38 pre‐validated targets. (4) After execution, the user can view a dashboard with results compared to a control for each target. (5) It is also possible to inspect the best pose of each ligand against each target and (6) download all files and execution logs.

The login and registration screens adhere to common web software standards. Users need an email address to register and will receive a confirmation email upon registration. Users can create an account login and register a password by clicking on “Sign up”. Once logged in, the software offers two options for data processing against *P. falciparum*. The first option, “Plasmodium falciparum with redocking”, uses the positive control of the redocking process to compare the results. The second option, “Plasmodium falciparum without redocking”, does not have a positive control and has not been launched yet. The dashed red arrow indicates the options available in the current version. Currently, only “*P. falciparum*” is available. The Plasmodocking usage flow can be seen in Figure 1.

To illustrate the results, we present a use case involving metabolites from *Capirona macrophylla*, a tree species native to the Amazon forest. These molecules were identified by mass spectrometry from their purified extract by our research group at the Center for Studies of Biomolecules Applied to Health (CEBIO). The metabolites were then provided to our Laboratory of Bioinformatics and Medicinal Chemistry (LABIOQUIM) for *in silico* testing using Plasmodocking.

**FIGURE 2:**
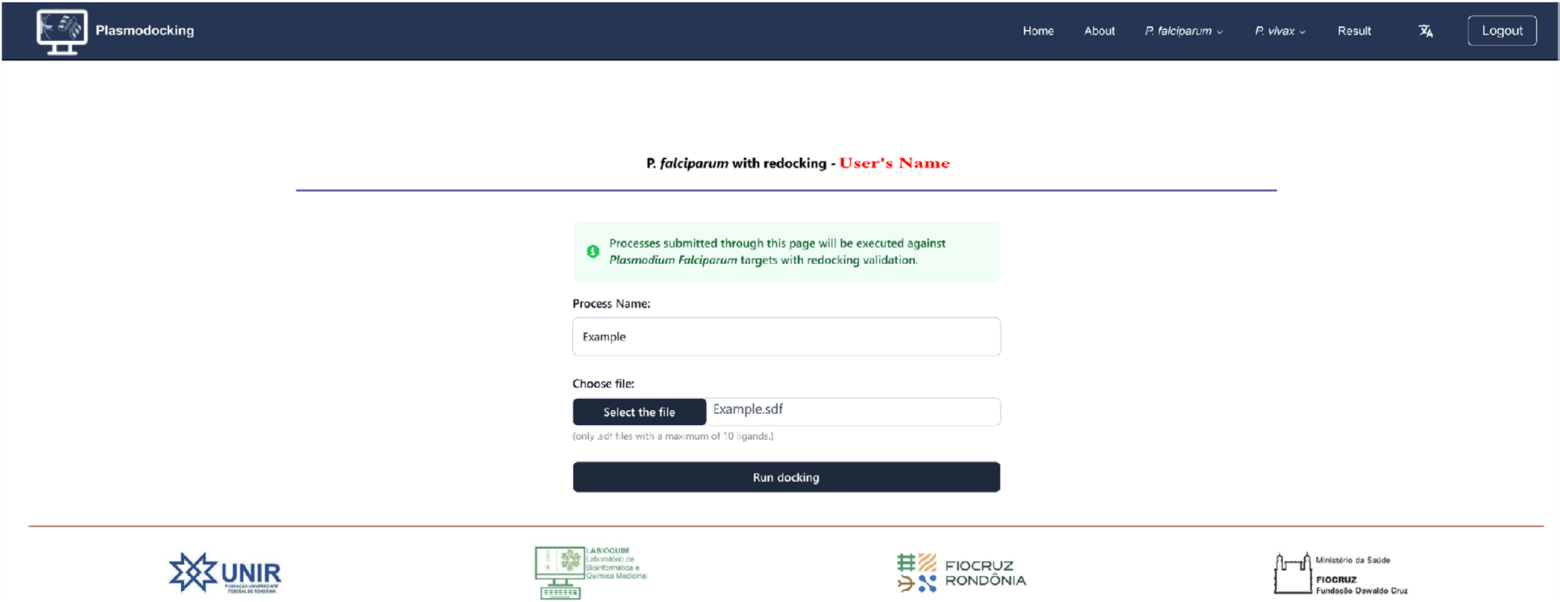
Submission screen for the *Example.sdf* file containing the five molecules tested in this case study.

To use the software, .*sdf* files of the three‐dimensional structures of the molecules to be tested are required. For this test, .*sdf* files of each of the five molecules selected from the mass spectrometry map were downloaded from the PubChem database [20]. These files were combined into a single .*sdf* file (Figure 2).

The metabolites were: Quinic acid (CID: 6508), Procyanidin B2 (CID: 122738), Neochlorogenic acid (CID: 5280633), Cryptochlorogenic acid (CID: 9798666) and 3‐O‐Feruloylquinic acid (CID: 9799386). Next, it is necessary to fill in the name of the experiment, upload the .*sdf* file, and execute. It is possible to dock up to 10 molecules per experiment. All user processes can be viewed by clicking on the “Result” link. In this interface, it is possible to download, delete, and analyze the results. Users can delete, download, or analyze the results of each process individually by navigating to the detailed view through the “Result” button.

**FIGURE 3:**
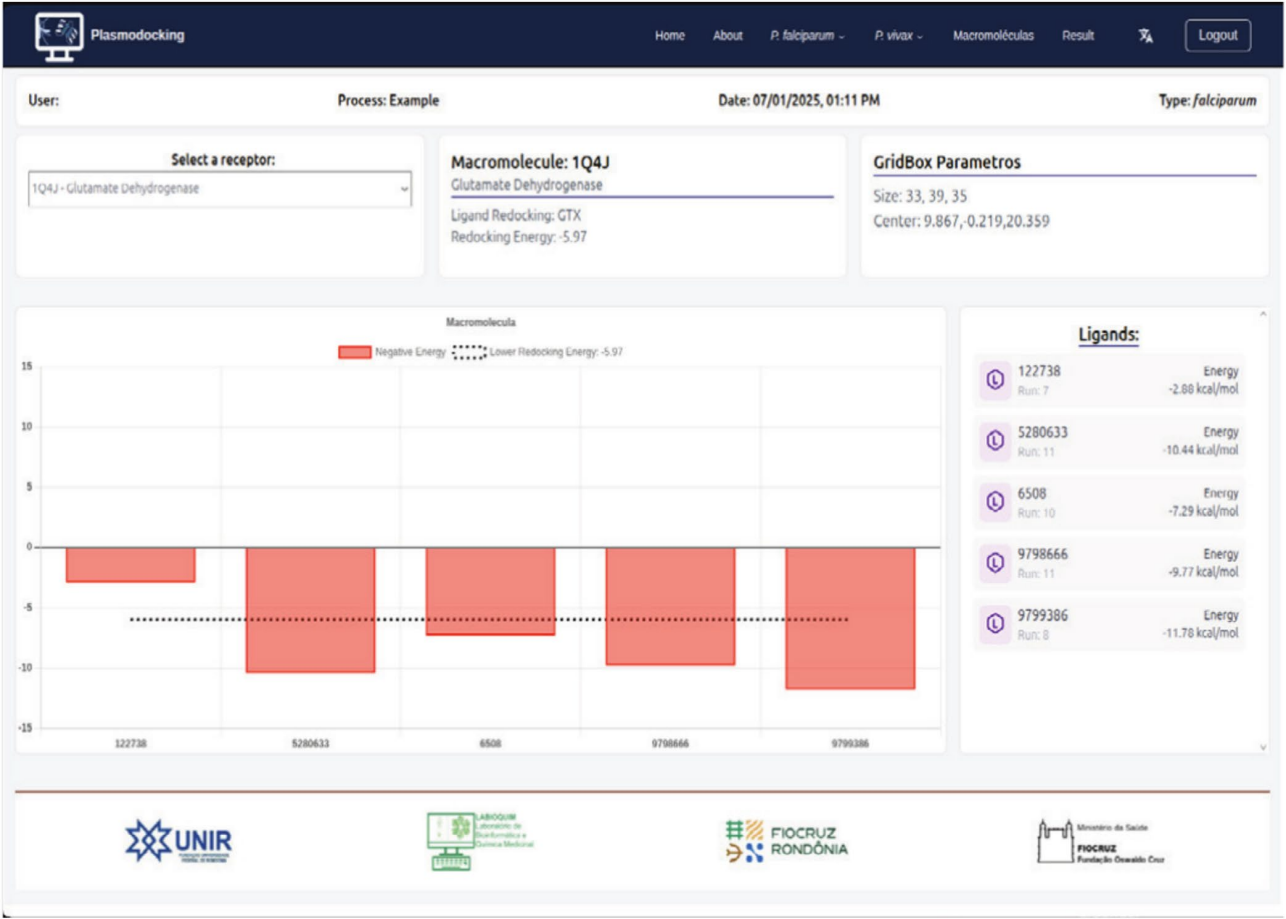
Panel showing results for the five molecules tested against the target macromolecule 1Q4J. The dashed line represents the redocking energy value = −5.97 kcal/mol. For this target, the best molecule was Neochlorogenic acid (PubChem CID: 5280633) with an energy of −11.78 kcal/mol.

The dashboard shows the energy cutoff, representing the positive control of the results, allowing a quick analysis of the ligands tested in relation to their inhibitors or crystallized ligands (Figure 3).

The dashboard shows results for the five molecules tested against 1Q4J macromolecule. On this screen, it is possible to select any of the 38 receptors, check the energy information, and the name of the ligand in the redocking process to the selected receptor. The accompanying table lists the energies associated with each of the molecules tested. The molecule with the lowest energy value was PubChem CID: 5280633 (Neochlorogenic acid), at −11.78 kcal/mol. The best energies for each of the five molecules tested in the case study are shown in Table 2. This allows the researcher to check which is the best (or most likely) receptor among the 38 available for each ligand. The best results were those that presented a greater variation in the energy of the candidate ligand and the redocking energy (ΔΔG).

**TABLE 2:** Best results for the five molecules tested in the case study.

Ligand name (Pubchem CID)	Best receptor (PDB Id)	ΔG redocking (kcal/mol)	ΔG ligand (kcal/mol)	ΔΔG^a^ (kcal/mol)
3‐O‐Feruloylquinic acid (9799386)	3SL1	−4.79	−12.07	−7.28
Neochlorogenic acid (5280633)	3SL1	−4.79	−11.21	−6.42
Cryptochlorogenic acid (9798666)	3SL1	−4.79	−11.18	−6.39
Quinic acid (6508)	1LYX	−2.40	−8.66	−6.26
Procyanidin B2 (122738)	6JW9	−4.88	−9.08	−4.20

^a^ ΔΔG is (ΔG ligand – ΔG redocking).

Observing the results in Table 2, it can be stated that the ligands 3‐O‐feruloylquinic acid (PubChem CID 9799386), neochlorogenic acid (PubChem CID 5280633) and cryptochlorogenic acid (PubChem CID 9798666) had Arginase (PDB id: 3SL1) as their best receptor. On the other hand, the ligands Quinic acid (PubChem CID 6508), and Procyanidin B2 (PubChem CID 122738) can be inhibitors of the Triosephosphate Isomerase (PDB id:1LYX), and Cysteine Protease Falcipain‐2 (PDB id: 6JW9) receptors as they have an interaction energy lower than the binding energy of the crystallized ligand. Data on the interactions of all five ligands against the 38 receptors can be found in the Supporting Information, which generates a total of 190 results (5 ligands × 38 receptors) (Table S1). This virtual screening took a little over 14 min.

## 3 Conclusions

Plasmodocking has proven to be a fast and effective tool for virtual screening of new ligands and identifying new molecular targets of *P. falciparum*. Users do not need to prepare the receptor or validate the best screening parameters, including the dimensions of the simulation grids. By simply submitting an .sdf file containing the candidate molecules, Plasmodocking performs all the energy calculations and presents a report, allowing the download of the best poses for each molecular target separately.

Currently, out of the 38 proteins already included, only 7 do not achieve an RMSD equal to or less than 2.00 Å. Additionally, the protein 5BOO (Dihydroorotate dehydrogenase) has been included with two inhibitor sites: the active site and the allosteric site according to a study by Margaret A. Phillips et al. [21]. It is worth noting that Plasmodocking keeps up with the evolution of the PDB, and all new receptors that appear will be added by the LABIOQUIM team.

## Author Contributions

Fernando Berton Zanchi and Joseph Albert Medeiros Evaristo formulated the idea and designed the flow and visual. Eduardo Pantoja de Macedo and Fernando Berton Zanchi developed the code. Fernando Loza Guariero and Fernando Berton Zanchi modeled and prepared the macromolecules and ligands, performed the redocking experiments, and established the minimum parameters. Elise Bittencourt de Laia, Geisa Paulino Caprini Evaristo, and Joseph Albert Medeiros Evaristo provided the biodiversity molecules, tested the interface with many other molecules, and revised the paper. Fernando Loza Guariero and Fernando Berton Zanchi wrote and revised the paper. All authors read and approved the final manuscript.

## Conflicts of Interest

The authors declare no conflicts of interest.

## Supporting information


**Table S1:** Best docking energies of the five *Capirona macrophylla* molecules against the 38 *Plasmodium falciparum* molecular targets available in Plasmodocking in order of ΔΔG. This result can be downloaded in .csv format directly from the results panel.

## Data Availability

The case study data are included in the manuscript and in the Supporting Information. The source code for Plasmodocking is freely available on GitHub https://github.com/LABIOQUIM/PlasmoDocking‐Client. Any further information is available from the corresponding author upon request.
