# Supplementary material for: PlasmoDocking: A User‐Friendly Open‐Source Web Tool for Virtual Screening Targeting Plasmodium falciparum Enzymes
Source: J Comput Chem. 2025 Sep 4;46(23):e70225. doi: 10.1002/jcc.70225 (PMC12410038; doi:10.1002/jcc.70225)
Supplement: Supplementary file 1 — Table S1: Best docking energies of the five Capirona macrophylla molecules against the 38 Plasmodium falciparum molecular targets available in Plasmodocking in order of ΔΔG. This result can be downloaded in .csv format directly from the results panel. [file JCC-46-0-s001.docx]

**Supplemental table 1.** Best docking energies of the five *Capirona macrophylla* molecules against the 38 *Plasmodium falciparum* molecular targets available in Plasmodocking in order of ΔΔG. This result can be downloaded in .csv format directly from the results panel.

| **PDB Id** | **Redocking energy [kcal/mol]** | **Cid Pubchem** | **Best Energy [kcal/mol]** | **ΔΔG^a^ [kcal/mol]** |
| --- | --- | --- | --- | --- |
| 1CJB | -4,89 | 6508 | -9,03 | -4,14 |
|  |  | 9799386 | -8,08 | -3,19 |
|  |  | 5280633 | -4,96 | -0,07 |
|  |  | 9798666 | -3,33 | 1,56 |
|  |  | 122738 | 354,71 | 359,60 |
| 1G1G | -8,57 | 5280633 | -10,96 | -2,39 |
|  |  | 9799386 | -9,5 | -0,93 |
|  |  | 9798666 | -8,53 | 0,04 |
|  |  | 6508 | -7,71 | 0,86 |
|  |  | 122738 | 62178,71 | 62187,28 |
| 1LF2 | -9,75 | 122738 | -10,59 | -0,84 |
|  |  | 5280633 | -10,15 | -0,40 |
|  |  | 9798666 | -9,18 | 0,57 |
|  |  | 9799386 | -8,57 | 1,18 |
|  |  | 6508 | -6,76 | 2,99 |
| 1LYX | -2,40 | 6508 | -8,66 | -6,26 |
|  |  | 5280633 | -5,4 | -3,00 |
|  |  | 9798666 | -2,82 | -0,42 |
|  |  | 9799386 | 6,1 | 8,50 |
|  |  | 122738 | 235304,72 | 235307,12 |
| 1P9B | -8,48 | 9798666 | -14,18 | -5,70 |
|  |  | 9799386 | -13,34 | -4,86 |
|  |  | 5280633 | -13,25 | -4,77 |
|  |  | 6508 | -8,78 | -0,30 |
|  |  | 122738 | 1,71 | 10,19 |
| 1Q4J | -5,97 | 9798666 | -11,3 | -5,33 |
|  |  | 9799386 | -10,58 | -4,61 |
|  |  | 5280633 | -10,44 | -4,47 |
|  |  | 6508 | -7,3 | -1,33 |
|  |  | 122738 | -6,35 | -0,38 |
| 1T26 | -4,64 | 6508 | -5,24 | -0,60 |
|  |  | 9799386 | 26,09 | 30,73 |
|  |  | 9798666 | 41,6 | 46,24 |
|  |  | 5280633 | 50,21 | 54,85 |
|  |  | 122738 | 8281,69 | 8286,33 |
| 1VYQ | -8,62 | 122738 | -10,78 | -2,16 |
|  |  | 9798666 | -10,41 | -1,79 |
|  |  | 5280633 | -9,95 | -1,33 |
|  |  | 9799386 | -9,92 | -1,30 |
|  |  | 6508 | -7,38 | 1,24 |
| 2I7C | -15,11 | 9799386 | -13,87 | 1,24 |
|  |  | 9798666 | -13,55 | 1,56 |
|  |  | 5280633 | -13,12 | 1,99 |
|  |  | 6508 | -8,38 | 6,73 |
|  |  | 122738 | 25,38 | 40,49 |
| 2WWF | -7,55 | 9799386 | -10,18 | -2,63 |
|  |  | 5280633 | -8,57 | -1,02 |
|  |  | 6508 | -8,11 | -0,56 |
|  |  | 9798666 | -6,00 | 1,55 |
|  |  | 122738 | 9169,1 | 9176,65 |
| 3BWK | -9,38 | 9799386 | -10,02 | -0,64 |
|  |  | 122738 | -9,02 | 0,36 |
|  |  | 9798666 | -8,93 | 0,45 |
|  |  | 5280633 | -8,73 | 0,65 |
|  |  | 6508 | -6,43 | 2,95 |
| 3PR3 | -4,99 | 9799386 | -9,01 | -4,02 |
|  |  | 9798666 | -8,88 | -3,89 |
|  |  | 5280633 | -8,09 | -3,10 |
|  |  | 6508 | -7,01 | -2,02 |
|  |  | 122738 | 8,72 | 13,71 |
| 3QGT | -8,53 | 9799386 | -11,45 | -2,92 |
|  |  | 5280633 | -9,86 | -1,33 |
|  |  | 9798666 | -8,76 | -0,23 |
|  |  | 6508 | -8,40 | 0,13 |
|  |  | 122738 | 7313,69 | 7322,22 |
| 3QS1 | -11,15 | 5280633 | -9,57 | 1,58 |
|  |  | 9798666 | -9,04 | 2,11 |
|  |  | 9799386 | -8,90 | 2,25 |
|  |  | 122738 | -7,38 | 3,77 |
|  |  | 6508 | -6,27 | 4,88 |
| 3QVI | -5,15 | 5280633 | -6,77 | -1,62 |
|  |  | 9799386 | -5,54 | -0,39 |
|  |  | 9798666 | -5,34 | -0,19 |
|  |  | 122738 | -5,16 | -0,01 |
|  |  | 6508 | -4,92 | 0,23 |
| 3SL1 | -4,79 | 9799386 | -12,07 | -7,28 |
|  |  | 5280633 | -11,21 | -6,42 |
|  |  | 9798666 | -11,18 | -6,39 |
|  |  | 6508 | -7,59 | -2,80 |
|  |  | 122738 | 872,7 | 877,49 |
| 3UJ8 | -9,82 | 9799386 | -14,26 | -4,44 |
|  |  | 9798666 | -12,8 | -2,98 |
|  |  | 5280633 | -12,78 | -2,96 |
|  |  | 6508 | -8,80 | 1,02 |
|  |  | 122738 | 26,98 | 36,80 |
| 3VI2 | -4,51 | 9799386 | -9,77 | -5,26 |
|  |  | 9798666 | -8,90 | -4,39 |
|  |  | 5280633 | -8,65 | -4,14 |
|  |  | 6508 | -6,71 | -2,20 |
|  |  | 122738 | 94,58 | 99,09 |
| 4J56 | -13,67 | 9798666 | -12,72 | 0,95 |
|  |  | 9799386 | -12,70 | 0,97 |
|  |  | 5280633 | -11,84 | 1,83 |
|  |  | 6508 | -7,72 | 5,95 |
|  |  | 122738 | 14,97 | 28,64 |
| 4J75 | -13,07 | 5280633 | -12,18 | 0,89 |
|  |  | 9799386 | -12,18 | 0,89 |
|  |  | 9798666 | -11,99 | 1,08 |
|  |  | 122738 | -9,88 | 3,19 |
|  |  | 6508 | -7,87 | 5,20 |
| 4JFA | -7,25 | 6508 | -8,00 | -0,75 |
|  |  | 9799386 | 0,13 | 7,38 |
|  |  | 9798666 | 0,24 | 7,49 |
|  |  | 5280633 | 0,31 | 7,56 |
|  |  | 122738 | 17057033,88 | 17057041,13 |
| 4PG3 | -9,19 | 9798666 | -13,74 | -4,55 |
|  |  | 9799386 | -13,21 | -4,02 |
|  |  | 5280633 | -12,78 | -3,59 |
|  |  | 6508 | -7,73 | 1,46 |
|  |  | 122738 | 50,03 | 59,22 |
| 4TR9 | -5,39 | 9799386 | -8,96 | -3,57 |
|  |  | 5280633 | -8,26 | -2,87 |
|  |  | 9798666 | -7,38 | -1,99 |
|  |  | 6508 | -6,69 | -1,30 |
|  |  | 122738 | 30,17 | 35,56 |
| 4ZCS | -9,68 | 9798666 | -12,34 | -2,66 |
|  |  | 122738 | -10,84 | -1,16 |
|  |  | 5280633 | -10,74 | -1,06 |
|  |  | 9799386 | -10,71 | -1,03 |
|  |  | 6508 | -7,98 | 1,70 |
| 5BOO | -8,72 | 6508 | -8,95 | -0,23 |
|  |  | 5280633 | 17,14 | 25,86 |
|  |  | 9799386 | 23,15 | 31,87 |
|  |  | 9798666 | 30,66 | 39,38 |
|  |  | 122738 | 723,10 | 731,82 |
| 5BOO^b^ | -9,54 | 9799386 | -12,85 | -3,31 |
|  |  | 5280633 | -12,62 | -3,08 |
|  |  | 9798666 | -11,50 | -1,96 |
|  |  | 6508 | -7,98 | 1,56 |
|  |  | 122738 | 45,27 | 54,81 |
| 5JAZ | -9,60 | 5280633 | -10,63 | -1,03 |
|  |  | 9798666 | -10,10 | -0,50 |
|  |  | 9799386 | -9,13 | 0,47 |
|  |  | 6508 | -6,56 | 3,04 |
|  |  | 122738 | -3,67 | 5,93 |
| 6FBA | -6,13 | 5280633 | -11,12 | -4,99 |
|  |  | 9799386 | -10,41 | -4,28 |
|  |  | 9798666 | -9,93 | -3,80 |
|  |  | 6508 | -7,87 | -1,74 |
|  |  | 122738 | 72,08 | 78,21 |
| 6JW9 | -4,88 | 5280633 | -9,63 | -4,75 |
|  |  | 9799386 | -9,39 | -4,51 |
|  |  | 122738 | -9,08 | -4,20 |
|  |  | 9798666 | -8,97 | -4,09 |
|  |  | 6508 | -7,01 | -2,13 |
| 6R8G | -6,55 | 5280633 | -10,50 | -3,95 |
|  |  | 9799386 | -9,37 | -2,82 |
|  |  | 6508 | -8,49 | -1,94 |
|  |  | 9798666 | -7,17 | -0,62 |
|  |  | 122738 | 514,33 | 520,88 |
| 7DIA | -5,88 | 9798666 | -6,52 | -0,64 |
|  |  | 9799386 | -6,49 | -0,61 |
|  |  | 122738 | -6,07 | -0,19 |
|  |  | 5280633 | -5,91 | -0,03 |
|  |  | 6508 | -5,76 | 0,12 |
| 7DPI | -9,66 | 122738 | -9,64 | 0,02 |
|  |  | 9799386 | -9,23 | 0,43 |
|  |  | 9798666 | -9,06 | 0,60 |
|  |  | 5280633 | -8,24 | 1,42 |
|  |  | 6508 | -6,82 | 2,84 |
| 7MXY | -7,01 | 9798666 | -11,67 | -4,66 |
|  |  | 9799386 | -11,31 | -4,30 |
|  |  | 5280633 | -10,78 | -3,77 |
|  |  | 6508 | -8,37 | -1,36 |
|  |  | 122738 | 801,97 | 808,98 |
| 7QB7 | -13,11 | 9798666 | -9,84 | 3,27 |
|  |  | 9799386 | -9,69 | 3,42 |
|  |  | 5280633 | -9,42 | 3,69 |
|  |  | 6508 | -7,51 | 5,60 |
|  |  | 122738 | -7,18 | 5,93 |
| 7ROR | -10,53 | 5280633 | -12,32 | -1,79 |
|  |  | 9798666 | -12,13 | -1,60 |
|  |  | 9799386 | -11,51 | -0,98 |
|  |  | 6508 | -8,21 | 2,32 |
|  |  | 122738 | 42,77 | 53,30 |
| 7TBC | -12,64 | 9798666 | -10,47 | 2,17 |
|  |  | 9799386 | -10,46 | 2,18 |
|  |  | 5280633 | -9,96 | 2,68 |
|  |  | 6508 | -8,20 | 4,44 |
|  |  | 122738 | 3,88 | 16,52 |
| 7ZGS | -5,04 | 9799386 | -6,54 | -1,50 |
|  |  | 6508 | -6,46 | -1,42 |
|  |  | 9798666 | -6,04 | -1,00 |
|  |  | 5280633 | -5,84 | -0,80 |
|  |  | 122738 | 1,75 | 6,79 |
| 8EWZ | -7,19 | 9798666 | -10,48 | -3,29 |
|  |  | 9799386 | -10,38 | -3,19 |
|  |  | 5280633 | -10,28 | -3,09 |
|  |  | 6508 | -8,31 | -1,12 |
|  |  | 122738 | 6,20 | 13,39 |

^a^ΔΔG is (ΔG ligand – ΔG redocking)

^b^Redocking in allosteric site.
